# EDUCATIONAL SERIES ON THE SPECIALIST VALVE CLINIC: How to run a specialist valve clinic: a collaborative series from the British Heart Valve Society and the British Society of Echocardiography

**DOI:** 10.1530/ERP-19-0035

**Published:** 2019-05-31

**Authors:** John B Chambers, Richard P Steeds

**Affiliations:** 1 Cardiothoracic Centre, Guy’s and St Thomas’ Hospitals, London, UK; 2 University Hospital Birmingham, Birmingham, UK; 3 Institute of Cardiology, University of Birmingham, Birmingham, UK

**Keywords:** valve disease, valve clinic

## Abstract

As heart valve disease increases in prevalence in an ageing population, comorbidities make patients increasingly hard to assess. Specialist competencies are therefore increasingly important to deliver best practice in a specialist valve clinic and to make best advantage of advances in percutaneous and surgical interventions. However, patient care is not improved unless all disciplines have specialist valve competencies, and there is little guidance about the practical details of running a specialist valve clinic. In this issue of *Echo Research and Practice*, the British Heart Valve Society (BHVS) and the British Society of Echocardiography (BSE) introduce a series of articles to guide all disciplines in how to run a valve clinic.

Valve disease is increasing in prevalence as our population ages (1) and is commonly regarded as the next cardiac epidemic (2). Patients are increasingly hard to assess as a result of comorbidities, while more is feasible in terms of percutaneous and surgical intervention. It is therefore less acceptable than ever for patients to be cared for by surgeons, physicians, nurses and physiologists/scientists without specialist competencies in heart valve disease. Standard best practice is now to assess and follow patients in a specialist valve clinic (3). The specialists in this clinic are also responsible for inpatient care, for managing programmes to improve the detection of valve disease, to train health-workers and to educate patients (4). In the United Kingdom, responsibility for the surveillance of patients before valve surgery may be transferred to scientists in a sonographer-led clinic and, after surgery, to a nurse (5).

It is not enough, however, simply to designate an outpatient clinic or an echocardiography list as valve focused. Although this can improve the efficiency of processes (6), the care of patients is not improved unless all disciplines have specialist valve competencies. The requirements for demonstrating competencies in valve disease (7) include training, volume of valve specific activity and (crucially) CPD. These are listed on the BHVS website (www.bhvs.org.uk) which permits self-nomination and the description of individual valve-focused experience and activity.

However, there is little guidance about the practical details of running a specialist valve clinic. The British Heart Valve Society (BHVS) and the British Society of Echocardiography (BSE) have therefore collaborated to provide a series of articles to guide all disciplines in how to run a valve clinic.

In the first four reviews of this series, published in this issue of *Echo Research and Practice*, David Messika-Zeitoun *et al.* discuss the reasons a valve clinic is necessary (8), and Sanjeev Bhattacharyya *et al.* outlines the role and organisation of a specialist valve clinic (9). Practical guidance on the care of patients with heart valve disease is given by Erwin Donal et al. in an article on the timing of surgery (10), and John Chambers provides a guide to the history and examination in heart valve disease (11).

## Declaration of interest

The authors declare that there is no conflict of interest that could be perceived as prejudicing the impartiality of this editorial.

## Funding

This work did not receive any specific grant from any funding agency in the public, commercial or not-for-profit sector.
